# An acute in-patient psychiatric service for 16- to 17-year-old adolescents in the UK: a descriptive evaluation

**DOI:** 10.1192/pb.bp.114.050161

**Published:** 2016-10

**Authors:** Venu Duddu, Abdulhakim Rhouma, Masood Qureshi, Imran Bashir Chaudhry, Terry Drake, Altaf Sumra, Nusrat Husain

**Affiliations:** 1 Lancashire Care NHS Foundation Trust, UK; 2 University of Manchester, UK

## Abstract

**Aims and method** The need for an age-appropriate in-patient service for 16- to 17-year-olds led to the development of a 6-bed acute admissions unit in a non-metropolitan county in the UK. We provide a descriptive evaluation of the first 2 years of its operation. All admissions from April 2010 to March 2012 were reviewed, clinical details systematically recorded and descriptively analysed.

**Results** Ninety-seven young people were admitted during this period (a third were compulsorily detained under the Mental Health Act 1983). The average length of stay was 3–4 weeks. The most common presenting complaints were self-harm and low mood, usually in the context of life events and childhood adversity. Nearly half had substance misuse and other risk-taking behaviours. A third presented with psychotic symptoms. Adjustment and anxiety disorders were most common, followed by alcohol/substance use disorders, depressive illnesses and psychotic illnesses. Comorbidity was the rule rather than the exception. Most patients improved by the time of discharge.

**Clinical implications** The unit provides an accessible and effective age-appropriate service and is likely to constitute an important component of the comprehensive child and adolescent mental health service strategy in the county.

The UK National Service Framework envisions the establishment of a comprehensive child and adolescent mental health service (CAMHS)^1^ and it has guided its development across the country into a four-tier structure. In-patient services, subsumed within tier 4, are specialist services that provide a therapeutic environment for young people with the most complex difficulties that cannot be managed safely in tier 3 community services.

Recent reviews of tier 4 CAMHS across the country have identified significant challenges related to increasing numbers of (emergency) referrals, a shortage of (developmentally appropriate) in-patient beds for 16- to 17-year-olds, an inability to deal with requests for urgent admissions in a timely fashion and concerns about the skills of adult in-patient wards in managing young people's difficulties.^2–6^ As a consequence, 16- and 17-year-olds who need emergency psychiatric admissions risk being denied admission or being inappropriately placed on adult psychiatric or medical wards. The need to address this issue became imminent in April 2010 due to the Mental Health Act 2007 amendment that required young people under 18 years of age who needed hospital admission for a mental disorder to be accommodated in an environment that is suitable for their age (and subject to their needs).

Different regions have adopted different solutions to meet this challenge. Some have extended existing units to accommodate young people up to 18 years of age. However, the forthcoming extension of the CAMHS age range up to 18 years is likely to have implications as the severity of illness and associated challenging behaviour of older adolescents may have a negative impact on younger children in the unit. Furthermore, the unit might begin to have a closer interface with local tier 3 CAMHS than with adult community teams. Some have developed transitional intensive support/assertive outreach services to manage this population in the community,^8^ whereas others have opted to commission beds from private providers. Some regions have also identified ‘age-appropriate’ adult wards for emergency adolescent admissions. In part, the types of solutions adopted have depended on local commissioning and financial imperatives, but also on the perceived magnitude of need and appetite to develop a comprehensive CAMHS in the region.^8^

This article describes the approach used in one county to address the mental health needs of 16- to 17-year-olds, and attempts a descriptive evaluation of its early experiences. Although ethical review and individual informed consent were not sought, the evaluation was considered and ratified by the trust's audit department.

## Method

### Setting

The setting is a non-metropolitan county in the UK with a population of around 1.45 million. The local mental health trust is commissioned to provide many tier 3 and tier 4 CAMHS, as well as adult mental health services. Tier 4 CAMHS (up to the age of 16) are commissioned by the regional specialised commissioning team (on behalf of the local primary care trusts), whereas services for 16- to 17-year-olds are included as a part of ‘block’ adult service contracts by individual primary care trusts. In anticipation of the age-appropriate statutory requirements, the trust developed a county-wide 6-bed acute in-patient psychiatric unit for this patient group in April 2010. Historically, young people have been known to ‘slip through the net’ and be passed from and to different services after reaching their 16th birthday. This unit was adopted (rather than other service developments) as it provides greater continuity of care and a more seamless approach.^9^

The unit is staffed by a consultant psychiatrist (half-time), a specialty doctor, an occupational therapist, a family therapist (both part-time), a psychologist (sessional), a dietician (sessional) and a full complement of nursing and healthcare support staff. It is supported by a tier 4 outreach team of CAMHS workers who are involved in pre-admission assessments and post-discharge care for up to 6 weeks. The team support the young people by providing care coordinators that attend all reviews and care programme approach (CPA) meetings and provide specialist therapeutic input. From an operational perspective, the unit accepts acute and emergency psychiatric admissions around the clock. Primary eating disorders and intellectual disabilities were excluded, unless the main focus was a mental health-related crisis. There are separate commissioning arrangements with identified private providers for young people with primary eating disorders.

Pre-admission assessments are carried out by mental health staff (usually nurses, social workers and less frequently, occupational therapists) from adult psychiatric gatekeeping teams (adult crisis resolution and home treatment (CRHT) teams and allied accident and emergency (A&E) liaison and criminal justice liaison teams) in a range of community settings (including hospital A&E departments, custody suites and patients' homes). During the day, these assessments are assisted by the CAMHS tier 4 outreach team nurses.

This article is an evaluation of the clinical experiences on this unit within the first 2 years of its existence and its impact on the wider tier 4 CAMHS service need in the county.

### Study

Case notes of all admissions (*n* = 97) to the unit over a 2-year period from April 2010 to March 2012 were reviewed retrospectively. A standard form was used to systematically record the following information: age, gender, area of residence, presenting complaints, salient developmental history (personal and family history), diagnosis, comorbidity, duration of in-patient stay, details of in-patient assessments, aftercare arrangements and discharge accommodation. Clinical severity and change on discharge (using the Clinical Global Impression Scale^10^) was recorded for the initial 41 consecutively admitted patients. Data were analysed descriptively and reported as frequencies and percentages.

To ascertain the impact of this unit on the service need for young people in the county, data were gathered on the number of admissions to adult wards in the 10 months leading up to April 2010, and also the number of admissions to adult wards between April 2010 and March 2012.

## Results

### Demographics and presenting symptoms

Of the 97 young people admitted to the unit during the study period, 50 were received in the first year: 58 (59.8%) were 17 years old, 44 (45.4%) were male and all but 1 were White British. Twenty-two (22.7%) had more than one admission to the unit (re-admissions). The average length of stay during the first year was 30 days (excluding one patient who had a 364-day admission), and 23.1 days in the second year. All were emergency admissions and had been ‘gatekept’ by the CRHT. Nine (9.3%) were admitted compulsorily under the Mental Health Act and a further 21 (21.7%) were detained after their admission.

### Assessment, management and diagnosis

The most frequent presenting complaints are outlined in Table 1. Sixty-eight per cent were admitted after an overdose or other self-harming behaviours, 55.7% had low mood and depressive symptoms and 29.9% presented with psychotic symptoms or paranoia.

**Table 1:** Most frequent presenting complaints, primary diagnoses and comorbidity^a,b^

Presenting complaints	Primary diagnoses	Comorbid conditions
Self-harm and/or overdose (68%)	Adjustment disorder, anxiety disorders, PTSD, social phobia (32.6%)	Maladaptive coping strategies, emerging personality difficulties (23.7%)

Low mood, depressive symptoms (55.7%)	Emerging personality traits or disorders (15.8%)	Harmful use/dependence on alcohol or illicit substances, secondary psychiatric symptoms (16.5%)

Psychotic symptoms, voices and paranoia (29.9%)	Schizophrenia, unspecified psychosis, delusional disorder, acute psychotic episode (14.7%)	ADHD and residual symptoms (11.3%)

Aggression and violence (7.2%)	Dysthymia, depressive episodes and manic episodes (14.7%)	Pervasive developmental disorder, Asperger syndrome (9.3%)

Impulsivity (6.1%)	Harmful use/dependence on alcohol or illicit substances, secondary psychiatric symptoms (14.7%)	Intellectual disability (9.3%)

Mood fluctuations (6.1%)	Impulsive self-harm (Z-codes^c^) (2.1%)	Unspecified psychotic symptoms (6.2%)

Alcohol and drug misuse-related symptoms (4.1%)	Acute confusional state (1.0%)	Conduct disorder, dissocial aggressive traits (6.2%)

Anxiety symptoms (3%)	Incomplete assessments (4.2%)	Generalised anxiety disorder, PTSD, social phobia (5.2%)

Elated and manic symptoms (1%)		Eating disorder (3.1%)

Social anxiety (1%)		

ADHD, attention-deficit hyperactivity disorder; PTSD, post-traumatic stress disorder.

a. In reducing order of frequency.

b. Presenting complaints do not add up to 100% due to patients presenting with more than one complaint.

c. Z-codes are part of the ICD-10 Chapter XXI classification system.

A large proportion of young people reported adverse childhood experiences as contributing to their clinical distress. These included bullying and emotional abuse (28.9%), sexual abuse (19.6%) and physical abuse (8.2%). A smaller number reported other significant family events such as parental illness and deaths (7.2%) and interpersonal and relationship difficulties (10.3%) as contributing to their distress.

Nearly half of the young people (49.5%) acknowledged being involved in various risk-taking behaviours, for instance illicit substance misuse, sexual behaviours and breaking the law/criminal behaviours.

Sixty young people (61.9%) had a history of self-harm, of whom 41 self-harmed irregularly and 19 self-harmed regularly. Cutting and overdosing were the most common modes of self-harm reported.

In terms of contact with mental health services, 40 young people (41.2%) had previous contact with tier 3 CAMHS and 12 (12.4%) were currently being managed by the early intervention teams (EITs) and adult community mental health teams (CMHTs). Twenty-three (23.7%) were previously (or currently) under the care of Social Services.

Adjustment and anxiety disorders were the most frequent primary diagnoses in the study group (32.6%), but emerging personality traits/disorders, alcohol and illicit substance use disorders, mood disorders and psychotic illnesses were also well represented (Table 1). Nearly three-fourths of the sample had comorbid psychiatric, psychological or social difficulties, with 42 (43.3%) young people having two diagnoses, 22 (22.7%) having three diagnoses and 10 (10.3%) having four diagnoses. Common comorbidity included alcohol and illicit substance misuse, maladaptive coping and emerging personality difficulties, residual symptoms of childhood attention-deficit hyperactivity disorder (ADHD), developmental disorders and intellectual disabilities (Table 1).

All young people who were admitted to the unit were assessed by the nursing and medical team: 36 undertook a formal occupational therapy assessment, 26 attended family therapy sessions and 25 were formally assessed by the team psychologist. Recommendations from these assessments guided the care planning process. Finally, 65 young people were treated with psychotropic medications (28 with antipsychotics, 41 with antidepressants and 1 with an ADHD medication). The Clinical Global Impression Scale was used to assess severity and clinical improvement. The figures (Tables 2 and 3) show that all but 11 young people improved significantly by the time of discharge. There were five delayed discharges (due to accommodation issues) and two premature discharges (against medical advice).

**Table 2:** Clinical Global Impression severity scores on admission and on discharge

	Admission, *n* (%)	Discharge, *n* (%)
Normal – not at all ill	0	11 (26.8)

Borderline mentally ill	5 (12.2)	17 (41.5)

Mildly ill	8 (19.5)	4 (9.8)

Moderately ill	18 (43.9)	4 (9.8)

Markedly ill	7 (17.1)	1 (2.4)

Severely ill	3 (7.3)	4 (9.8)

**Table 3:** Clinical Global Impression improvement

	*n* (%)
Very much improved	2 (4.9)

Much improved	22 (53.7)

Minimally improved	6 (14.6)

No change	4 (9.8)

Minimally worse^a^	2 (4.9)

Very much worse^a^	5 (12.2)

a. Young people who became worse after admission were those who needed transfer to psychiatric intensive care unit.

Most young people (*n* = 58, 59.8%) were discharged to their homes, but 17 were discharged to various forms of supported accommodation (17.5%). Eight patients (8.2%) were deemed to be (or have become) too acutely unwell and needed to be transferred to a psychiatric intensive care unit (PICU), whereas five (5.1%) were transferred to other hospital wards (e.g. medical wards, adult psychiatry wards). Of the 8 young people transferred to the PICU, 4 needed to be subsequently transferred to a low secure/high-dependency unit for longer-term treatment for repetitive self-harm and emerging personality difficulties.

All the young people were referred to various adult community mental health teams (CMHTs and EITs), who supported them after discharge. The discharge care plans also included intensive short-term step-down support from the CRHTs and the tier 4 outreach team (the latter for up to 6 weeks).

### Impact of the unit on admissions to adult wards

The average bed occupancy at the unit in the second year was 76% (monthly range 47–95%). During the evaluation period, 8 young people were admitted to various adult psychiatric wards. Of these, 6 admissions were due to non-availability of beds at the unit (these were brief admissions that were transferred back to the unit as soon as an age-appropriate bed became available) and 2 were admitted to adult PICUs.

In the 10 months before the unit was opened, 36 young people were admitted to adult in-patient psychiatric wards. Self-harm, low mood and suicidal behaviours (*n* = 18), and voices, paranoia and psychotic symptoms (*n* = 13) constituted the most common presenting complaints.

## Discussion

The unit evaluated in this review complements an 8-bed planned admissions unit in the county which accepts young people up to the age of 16. Together, they constitute the in-patient components of the tier 4 CAMHS in the county.

The evaluation involves a retrospective analysis of all young people who were admitted over a 2-year period. As such, this is a comprehensive reflection of those young people who needed tier 4 in-patient services in the county. However, having been gleaned from case records, data are limited by the accuracy of the record-keeping, and are also subject to a degree of cognitive bias in the interpretation of the same. A prospective study utilising additional data pertaining to further demographic details of the cohort, severity and the nature of the illness could possibly overcome these issues. Also, qualitative reports of the views of both the young people and the clinician in the unit would be beneficial. Further, it is difficult to accurately extrapolate the findings to a county-wide service-need for this population as this evaluation has not included the entire sampling frame of all the young people who were assessed by gatekeeping teams during this time frame (and were deemed to not need in-patient care). Future research in this area could explore the outcomes obtained with other, wider tier 4 services in the county.

The unit's operational policy has identified broad principles (around clinical risk) to guide the assessment for suitability of admission, and as such, is not overly restrictive in its remit. This generated some anxieties as to whether the availability of the service would stimulate an increase in the number of admissions. Similar anxieties revolved around whether adult CRHTs could be reliably (and appropriately) gatekeeping these beds, and also whether a young people's transitional service model interfacing with different adult mental health services was feasible. The results of our evaluation do not appear to bear out these anxieties, as the average number of admissions in the first and second years is not significantly different from the numbers in the preceding 10 months. This also validates the stability of admission thresholds applied by CRHTs in conducting effective gatekeeping assessments, and in the role of tier 4 outreach teams in supporting this process.

Our gatekeeping procedure is quite different from our sister (planned admissions) CAMHS in-patient unit for 12- to 16-year-olds (located in Lancaster), where a detailed pre-admission assessment is conducted by the CAMHS team to ascertain suitability for in-patient stay. Adult CRHT (and allied liaison and criminal justice teams) assess young people for suitability for admission to the unit. This is a 24-hour service, with patients being assessed in various emergency situations including hospital A&Es, custody suites and patients' homes. Although our procedures might potentially increase the risk of inappropriate admissions (due to emergency assessments by non-CAMHS mental health professionals), they also afford the advantage of the service being more available and accessible around the clock. The risk of inappropriate admissions was not borne out in practice, in that all admitted patients did need a period of in-patient risk management and a more comprehensive assessment of their needs, which served as a basis for planning their future care. Social and accommodation difficulties did sometimes result in a more prolonged admission, but these were also (eventually) managed through close liaison with Social Services and housing agencies.

There is a significant clinical interface between a number of adult psychiatric and CAMHS clinical teams in providing care for 16- and 17-year-olds in the county. General practitioners refer young people (over 16 years of age) to adult primary care mental health teams (or the EITs if a psychosis is suspected), who act as the single point of access. Following an initial assessment, young people are then signposted to various secondary care services if clinically indicated. Patient care is guided by CPA principles, and young people are discharged from our in-patient unit to the care of various adult CMHTs (including the complex care and treatment teams (CCTT) and EITs). These (multiple and rather complex) clinical interfaces can result in the young person often having contact with a range of different community teams as their care progresses, and can potentially contribute to inconsistencies and discontinuities in care. Anecdotal evidence suggests that many young people found this stressful and overwhelming. In some instances, we have also come across some difficulties due to differing philosophical and clinical approaches to patient care among various adult psychiatric and child and adolescent mental health services. These focused mainly around CAMHS teams being perceived as more systemic and family oriented, with a greater emphasis on a collaborative/participatory approach to care compared with adult teams.

The clinical profile of our sample, that is the range of presenting complaints, history, diagnostic patterns and comorbidity, is reflective of a population of young people who have severe and complex mental health and social difficulties, and pose significant risks to themselves. As such, they seem to reflect the needs of 16- and 17-year-old tier 4 clients, as identified in Kurtz's review of the evidence base for tier 4 CAMHS.^6^

From an outcomes perspective, the majority of young people achieved symptom stabilisation and a reduction of distress (even during the relatively short admission), and were discharged home with mental health support. These findings served to allay some of our previous anxieties and highlighted that it is possible to develop a transitional service interfacing with various adult mental health teams. However, the forthcoming extension of the CAMHS age range up to 18 years is likely to have implications and the unit might begin to have a closer interface with local tier 3 CAMHS than with adult community teams.

Among the different models of in-patient adolescent care,^6^ ours focuses on an acute and intensive assessment, risk management and symptom stabilisation before rapid discharge. We came across another paper that reported on a similar model of service delivery.^11^ Although they were more inclusive in terms of the age range, the clinical profile of their patients, their average length of stay and outcomes are broadly comparable with our findings, thus reiterating the assertion that it is possible to develop a flexible, effective and efficient service for this patient population. The only other service evaluation that we came across was that by Cotgrove,^12^ but their service model was very different from ours, thus precluding any comparisons.

The absence of an age-appropriate PICU and a longer-term high-dependency therapeutic unit to support our work locally were important challenges necessitating liaison and close collaboration with individual primary care trust commissioners. Both these serve to emphasise that a unit like ours only constitutes a small component of a comprehensive tier 4 CAMHS, and is likely to work best in partnership with local commissioners, an effective outreach team, a PICU, a longer-term planned admissions unit (for those needing longer-term care), and an effective community service to provide support and aftercare for discharged patients.

Finally, it was found that 1 of the 97 young people admitted to the unit was from a Black and minority ethnic background, whereas county-wide that number is 9%. There could be several reasons for this and they may warrant further exploration.

We suspect that many of the challenges reported here are not limited to this particular county, and are a reflection of generally inconsistent and non-uniform commissioning arrangements for this patient group. It is likely that future reviews of the National Specialised Commissioning Team will help to iron out these inconsistencies and help in the development of a more uniform tier 4 commissioning strategy across the UK.
